# Programmable and Surface‐Conformable Origami Design for Thermoelectric Devices

**DOI:** 10.1002/advs.202309052

**Published:** 2024-01-02

**Authors:** Yue Hou, Zhaoyu Li, Ziyu Wang, Xingzhong Zhang, Yang Li, Chang Li, Haizhong Guo, Hongyu Yu

**Affiliations:** ^1^ Department of Mechanical and Aerospace Engineering The Hong Kong University of Science and Technology Kowloon Hong Kong SAR 999077 P. R. China; ^2^ HKUST Shenzhen‐Hong Kong Collaborative Innovation Research Institute Shenzhen 518048 P. R. China; ^3^ The Institute of Technological Sciences Wuhan University Wuhan 430072 P. R. China; ^4^ Key Laboratory of Artificial Micro‐ and Nano‐Structures of Ministry of Education School of Physics and Technology Wuhan University Wuhan 430072 P. R. China; ^5^ Key Laboratory of Material Physics Ministry of Education School of Physics and Microelectronics Zhengzhou University Zhengzhou 450052 P. R. China; ^6^ Collaborative Innovation Center of Light Manipulations and Applications Shandong Normal University Jinan 250358 P. R. China

**Keywords:** origami design, surface conformable, thermoelectric device

## Abstract

Thermoelectric devices (TEDs) show great potential for waste heat energy recycling and sensing. However, existing TEDs cannot be self‐adapted to the complex quadratic surface, leading to significant heat loss and restricting their working scenario. Here, surface‐conformable origami‐TEDs (o‐TEGs) are developed through programmable crease‐designed origami substrates and the screen‐printing TE legs. Compared with “π” structured TEDs, the origami design (with heat conductive materials) changed the heat‐transferring direction of the laminated TE legs, resulting in an enhancement in enlarging Δ*T*/*T*_Hot_ and *V*_out_ by 5.02 and 3.51 times. Four o‐TEDs with different creases designs are fabricated to verify the heat recycling ability on plane and central quadratic surfaces. Demonstrating a high *V*_out_ density (up to 0.98 ^−2^at Δ*T* of 50 K) and good surface conformability, o‐TEDs are further used in thermal touch panels attached to multiple surfaces, allowing information to be wirelessly transferred on a remote display via finger‐writing.

## Graphical abstract

Surface conformable origami designed TEGs (o‐TEGs) are presented in this paper. The programmble origami creases design allows a piece of unfolded paper‐based TEG to be folded to wrapped around quadratic heat source surfaces, e.g., cylinderical, conical, and hemispherical surfaces.

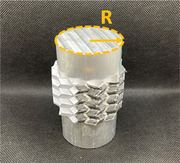

## 1 Introduction

Recent development in the Internet of Things (IoT) has heightened the need for a sustainable power supply of sensors. Thermoelectric (TE) devices, which can convert environmental heat energy into electricity, have become a promising solution for both power supplies for wireless sensing systems and sensors for physical motion monitoring.^[^ ^1^, ^2^, ^3^, ^4^, ^5^ ^]^ In life, heat sources that can be recycled usually have quadric surfaces. For example, hot water pipes process a cylindrical surface, while coffee cups and lamp shades share a conical surface. As heat conduction is the dominant factor in the heat transferring process, energy recovered by the TED strongly depends on a compact contact with the heat source surface. In addition, both voltage outputs (*V*_out_) and maximum power output (*P*_max_) of the TED have positive relations with the temperature difference across TE legs (Δ*T*).^[^ ^6^ ^]^ Thus, developing TEDs conforming to complex quadric surfaces with large Δ*T* is quite demanded.

To date, a number of research works have strived for better thermal contact. These methods can be categorized into either increasing the device flexibility^[^ ^7^, ^8^, ^9^, ^10^ ^]^ or directly forming shape‐matched TE materials according to the cylindrical surface of the heat source.^[^ ^11^, ^12^ ^]^ For the formal type, TE materials with low ductility, like rigid cuboids and films, are designed with stretchable substrates to pursue good contact with complex human skin surfaces.^[^ ^13^, ^14^, ^15^ ^]^ However, conformability is largely limited by the rigid cuboid size, and the air gap between the bottom cuboid and heat source surface will vastly decrease the output performance. Besides, few of the previously reported flexible TEDs have taken into account complex quadric surfaces other than cylindrical surfaces. Directly forming conformable shaped TE segments is another way to increase the TED performance. Current literature on 3D‐printed TED pays particular attention to fabricating structurally designed TE segments. J. Son and his group had directly printed the hemi‐ring or ring shape TE segments using inorganic Bi_2_Te_3_ particles‐based and PbTe particles‐based inks, respectively. Integrating electrodes and Ag epoxy solder, the printed TE segments could be assembled on the outer surface of a hot water pipe for energy recovery.^[^ ^16^, ^17^, ^18^ ^]^ Another way to form a hemi‐ring shape was directly bending the shape of an Ag_20_S_7_Te_3_ ingot strip to fit the target cylinder.^[^ ^11^ ^]^ So far, these directly formed rigid TE segments are solely targeted for a specific cylindrical tube with inalterable bottom radii. The extra copper electrodes and wires bonding processed by hand largely limited the number of TE pairs, affecting the whole output performance of TEDs.

Here, we reported plane and central quadratic surfaces conformable TEDs on origami‐structured paper substrates. Line creases within degree‐4 vertices origami can be programmable designed to ensure conformable contact with different targeting surfaces. In origami TEDs (o‐TEDs), the heat transferring direction within every TE segment goes along with the screen‐printed direction of TE laminate, thus achieving a large Δ*T* after folding along pre‐designed creases. Compared with the traditional “π” structure, the adapted standing‐up o‐TED in the same amount of TE materials demonstrated a huge increase in both *V*_out_ per area and *P*_max_ per area. The plane, cylindrical, conical, and hemispherical o‐TEDs with 24 TE pairs were successfully fabricated for different surfaces with good *V*_out_ density and *P*_out_ density, thus demonstrating the feasibility of origami design in shape conformable energy harvesting TEDs. Notably, plane and cylindrical o‐TEDs with first‐class *V*_out_ density per *K* in flexible TEDs (19.58 and 9.84 mV K^−1^ cm^2^) were further innovatively designed as shape‐conformable touch panels (TTPs), and corresponding circuit designs for wireless information transmission and remote display were also developed to track the finger‐moving path and demonstrate English letters on the computer, providing a practical application of the thermoelectric device for future flexible electronics.

## 2 Design and Fabrication of o‐TEGs

**Figure 1:**
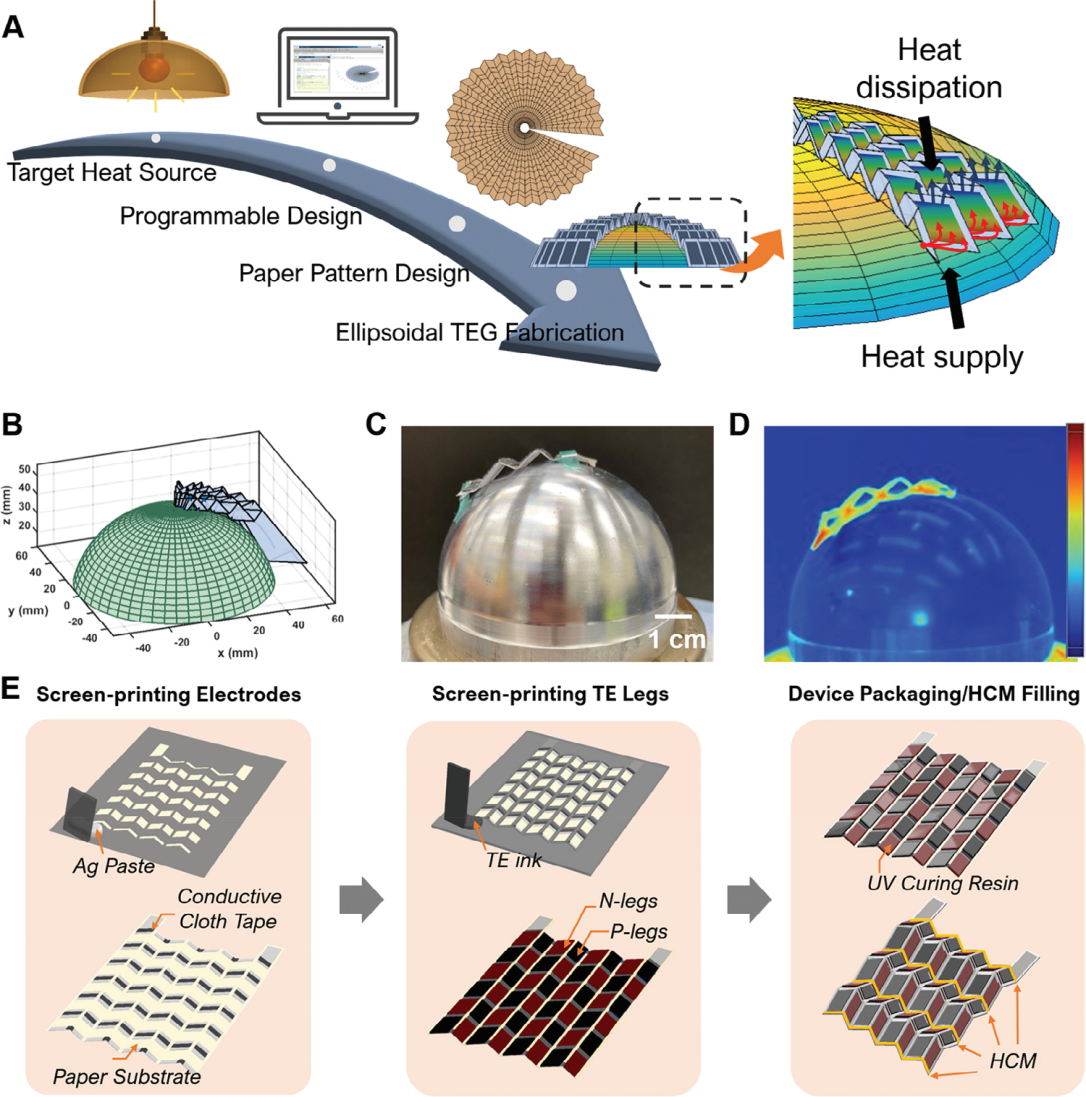
Design and fabrication of the o‐TED. A) The schematic diagram of the design process of o‐TED for an ellipsoidal lamp. B) Programmable origami structure design samples. C) Optical image for hemispherical surface adaptive o‐TED with 6 TE pairs. D) Infrared image for hemispherical surface adaptive o‐TED under the working scenario. E) The schematic diagram for the fabrication process of the o‐TED.

The advantages of using 3D origami structures fall on both the surface conformability and heat transfer aspects. As indicated in the designing scheme of **Figure** **1**A, for a customized design (here is an ellipsoidal lamp), when the section profile and the cap profile geometry are given, the programmable design help settle down the crease length and angles between creases. The design details are provided in the following section and Supporting Information. This process ensures that the folded paper substrate can fit well with the lamp surface. When the light turns on, the heat energy can be harvested from the bottom side of o‐TED through heat conduction. Meanwhile, as illustrated in the enlarged schematic image of Figure 1A, the 3D architecture ensures that the upper side is exposed to the ambient environment with relatively low temperature compared with the bottom side, thereby, ensuring the better performance of o‐TED based on the Seebeck effect. This design method applies to other complex quadratic surfaces, including cone, hemisphere, ellipsoid, and paraboloid surfaces (simulated figures are in Figure 1B; Figure S5, Supporting Information). The photo image and the infrared image during the working state of the folded o‐TED are demonstrated in Figure 1C,D. It can be found that the device is well attached to the hemispherical surface with the distinctive Δ*T* along each plane. To fabricate these o‐TEDs, the o‐TED with the traditional Miura‐ori pattern is taken as an example (Figure 1E). From the bottom up, the o‐TED comprises the paper substrate, electrodes, TE segments, packaging layers, and finally, the filling layer of the heat conductive materials (HCM). Since the heat transferring direction goes along the origami plane, the N‐type and P‐type segments are in series connection with only one layer of bottom electrodes. The silver paste was first screen‐printed on the paper, and another layer of laser‐cut conductive cloth tape was transferred to the crease region to avoid the fracture of the silver paste electrodes after folding. One thing worth mentioning is that the paper substrate was already folded to form the Miura‐ori shape memory creases before we started fabricating the electrodes. Then, N‐type and P‐type inks containing binder solvent, Bi_2_Te_2.7_Se_0.3_ or Bi_0.5_Sb_1.5_Te_3_ powder, were screen‐printed to form the TE pairs. A layer of UV‐cured resin was covered on top of the TE segments to work as the protecting layer. Notably, the resin layer also creates higher stiffness for the in‐plane area, facilitating the folding process afterward. After that, a thin layer of Parylene‐C was deposited on the surface of o‐TED for electrical insulation. To improve the heat conduction between the heat source and the bottom side of the TE segments, the HCM was squeezed out by a dispenser on the bottom side of the TE segments with zigzag routes (yellow lines in Figure 1E). Finally, the o‐TED was pressed by a pair of 3D‐printed molds to form the folded shape (Figure S6, Supporting Information).

## 3 Programmable Design for o‐TEDs

**Figure 2:**
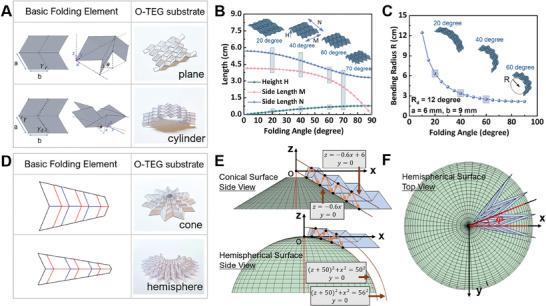
Programmable design for origami paper substrates. A) “One‐to‐many” design type for plane and cylindrical targeting surfaces. B) The relation between structure geometrical parameters (*H*, *M*, *N*) and different folding angles of the origami structure for plane heat source surface. C) The geometrical parameters (*R*) variation under different folding angles of the origami structure for cylindrical heat source surface. D) “One‐to‐one” design type for complex quadric surfaces, including conical and hemisphere surfaces. E) Side view of the folded pattern designs for conical and hemispherical surfaces. F) Top view of the folded pattern design for the hemispherical surface.

Programmable designs for paper substrates of o‐TED adapted to plane or central quadratic surfaces can be categorized into two types. The first type is “one to many,” meaning the one design crease pattern can be adapted to multiple heat source surfaces, including plane and cylindrical surface adapted o‐TEDs (shortly named as cylindrical o‐TED in the following contents). For plane o‐TED, three preset parameters (two side lengths *a*, *b* and their angle γ in **Figure** **2**A) are first settled down as 6 cm, 9 cm, and 60°. Then, as shown in Figure 2B, when increasing the folding angle θ for a 4 × 3 (*M* × *N*) array, the whole structure could shrink to a small size while remaining a plane‐adapted surface, which allows it to attach to a plane with a small surface area. The design process for the cylindrical o‐TED is updated on the plane o‐TED with the same corresponding side length and angle γ, where one more preset parameter, the deviation angle γ_d_, is needed. Here, the γ_d_ is set as 12^°^ in the following design. As illustrated in Figure 2C, when θ increases from 10^°^ to 90^°^, the corresponding cylinder radius (*R*) drops from 12.46 to 2.16 cm, which is within an acceptable range of the size of cylinder heat sources in our daily life. Under different θ, the adapted surface remains to be a cylinder with a smaller bottom circular radius, thus allowing one cylinder o‐TED to attach to multiple‐size cylinder surfaces.

The second programmable type is “one to one,” which covers all the central quadratic surface with the o‐TED wrapping direction along the generatrix (different from the cylinder o‐TED whose wrapping direction is along the peripheral direction, i.e., the direction perpendicular to the generatrix).^[^ ^18^ ^]^ For this type, the target surface parameters are first settled for designing the basic folding elements (Figure 2D). Here, the conical and hemispherical surfaces are taken as examples. The equation of the two parallel curves, including the generatrix curve, is derived for the targeting surface. All vertexes on the central line of the basic folding elements fall onto these two curves (Figure 2E), while the distance between these paralleled curves controls the size of basic folding elements. Another parameter that should be determined first is the angle *φ*, which will guide the wrapping density of the o‐TED (Figure 2F). More programmable details are presented in the Supporting Information.

## 4 Simulation and Experimental Results

**Figure 3:**
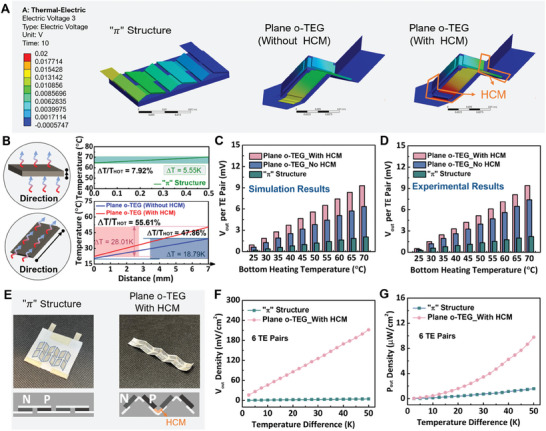
Effect of 3D structure design and HCM on the performance of o‐TED. A) Finite elements analysis (FEA) results in the electric voltage output of “π” structure (6 TE pairs), plane o‐TED with and without HCM (2 TE pairs) when the bottom heating temperature reaches 70^°^ C. B) Schematic diagram of the heat transferring direction of the “π” structure and o‐TEG. And FEA results of temperature profile of “π” structure, o‐TEG without HCM, and o‐TEG with HCM when the bottom heating temperature reaches 70 ^°^C. C) FEA; D) experimental results on the open‐circuit voltage output (*V*_out_) per TE pair. E) Optical images for the “*π*” structure and plane o‐TED with HCM (both with six TE pairs). Experimentally measured: F) *V*_out_ density; G) *P*_out_ density for both “π” structure TED and plane o‐TED with HCM.

According to *V*_out_  =   *N* × *S*_NP_ × Δ*T*, where *N*, *S*_NP_, Δ*T* are TE pair number, Seebeck coefficient of one TE pair, and temperature difference across TE legs, respectively. Therefore, the *V*_out_ has a positive relationship directly with the Δ*T*. In the preceding section, it was stated that the 3D architecture of the screen‐printed o‐TED contributes to improving its performance by creating a larger Δ*T*. For this reason, a finite element analysis (FEA) was conducted using ANSYS software's Steady‐state Thermal and Thermal‐electric modules to accurately forecast the ∆*T* and thermoelectric voltage output (*V*_out_) (**Figure** **3**A) between two sides of the TE segment. As shown in the 3D model design in Figure 3A and the optical images in Figure 3E, for both simulation and experimental testing, the TE element size is the same for both TED with “π” structure and plane o‐TED. As predicted in Figure 3B, when the bottom heating temperature reaches 70 ^°^C, the *T*_cold_ for “π” structure TED can reach 64.54 ^°^C on its upper side due to the direct contact on the heat source without a heat sink, thereby leading to a small Δ*T* of 5.55 ^°^C. However, for 3D shape o‐TED, *T*_cold_ entirely relies on the heat passing through the TE legs, which results in a small *T*_cold_ of 20.47 ^°^C with a larger Δ*T* of 18.79 ^°^C. In this circumstance, *T*_hot_ is still small due to the air gap between the bottom surface of TE segments and the heat source. Therefore, to better enlarge this value, HCM with good thermal conductivity has been added to form a heat conduction channel between the heat source and the bottom surface of TE segments, thereby, further enlarging the Δ*T* = 28.01 ^°^C. The experimental test on the thermal conductivity of the HCM was provided in the Supporting Information (Figure S7, Supporting Information). The maximum conversion efficiency η_*max*_ can be regarded as

(1) $$\;\eta_{max}=\frac{\Delta T}{T_{Hot}}\;\;\eta_{0}=\frac{T_{Hot}-T_{Cold}}{T_{Hot}}\;\frac{\sqrt{Z\bar{T}+1}-1}{\sqrt{Z\bar{T}+1}+T_{Cold}/T_{Hot}}$$

where η_0_ largely depends on the figure of merit ($Z\bar{T}$).^[^ ^8^, ^20^, ^21^ ^]^ From Figure 3B, the value of Δ*T*/*T*_Hot_ for these three devices are 7.92%, 47.86%, and 55.61%, respectively. Based on the temperature value in Figure 3B, the relationship between the η_*max*_ and the Z value can be drawn in Figure S14B (Supporting Information).

For all three structures, the *Z* value can be regarded as

(2) $$Z\;=\frac{S^{2}}{RK}\;=\frac{{\left(S_{P}-S_{N}\right)}^{2}}{\left(\frac{l_{P}}{A_{P}}\rho_{P}+\frac{l_{N}}{A_{N}}\rho_{N}\right)\left(\frac{A_{P}}{l_{P}}k_{P}+\frac{A_{N}}{l_{N}}k_{N}\right)}$$

which can be further estimated with the given value *S_P_*, *S_N_*, *k_P_*, *k_N_* ρ_*p*_, and ρ_*N*_. As detailly discussed and calculated in the Supporting Information, the *Z* value for these three types of structure is at the same order of magnitude with slight difference. Thus, based on the Figure S14B (Supporting Information), the superiority of 3D structure design and further enhancement of conversion efficiency with HCM can be verified. With the simulation results on *T*_cold_ and *T*_Hot_ in the aforementioned three circumstances, the *V*_out_ of these devices can be further simulated with the electric‐voltage module. The voltage output per TE pair for these three architectures are summarized and compared under different heating temperature from 25 ^°^C to 70 ^°^C. As illustrated in Figure 3C, single TE pair with and without HCM can achieve distinctively higher *V*_out_ of 9.30 and 6.34 mV when the bottom heating temperature reaches 70°C, which is 3.51 times and 2.07 times larger than that of “*π*” structure.

A similar trend can also be observed in the experimental testing of *V*_out_ for the TEDs with 6 TE pairs, and the testing results for V_out_ per TE pairs were collected in Figure 3D. The large *V*_out_ and *P*_max_ per area are also tested for TED with “π” structure and o‐TED (Figure 3E). As shown in Figure 3F,G, compared with the “π” structure one, the smaller effective area from the origami structure (0.234 cm^2^) leads to a more distinctive difference in *V*_out_ per area, which is 46.98 times larger than that of the “π” structure. For measuring, although the inner resistance for the 3D architecture is larger than the “π” structure (269.11 Ω for o‐TED and 17.30 Ω for the “π” structure), the testing results of *P*_max_ per area for o‐TED also demonstrate 6.18 times as large as that of the “π” structure.

**Figure 4:**
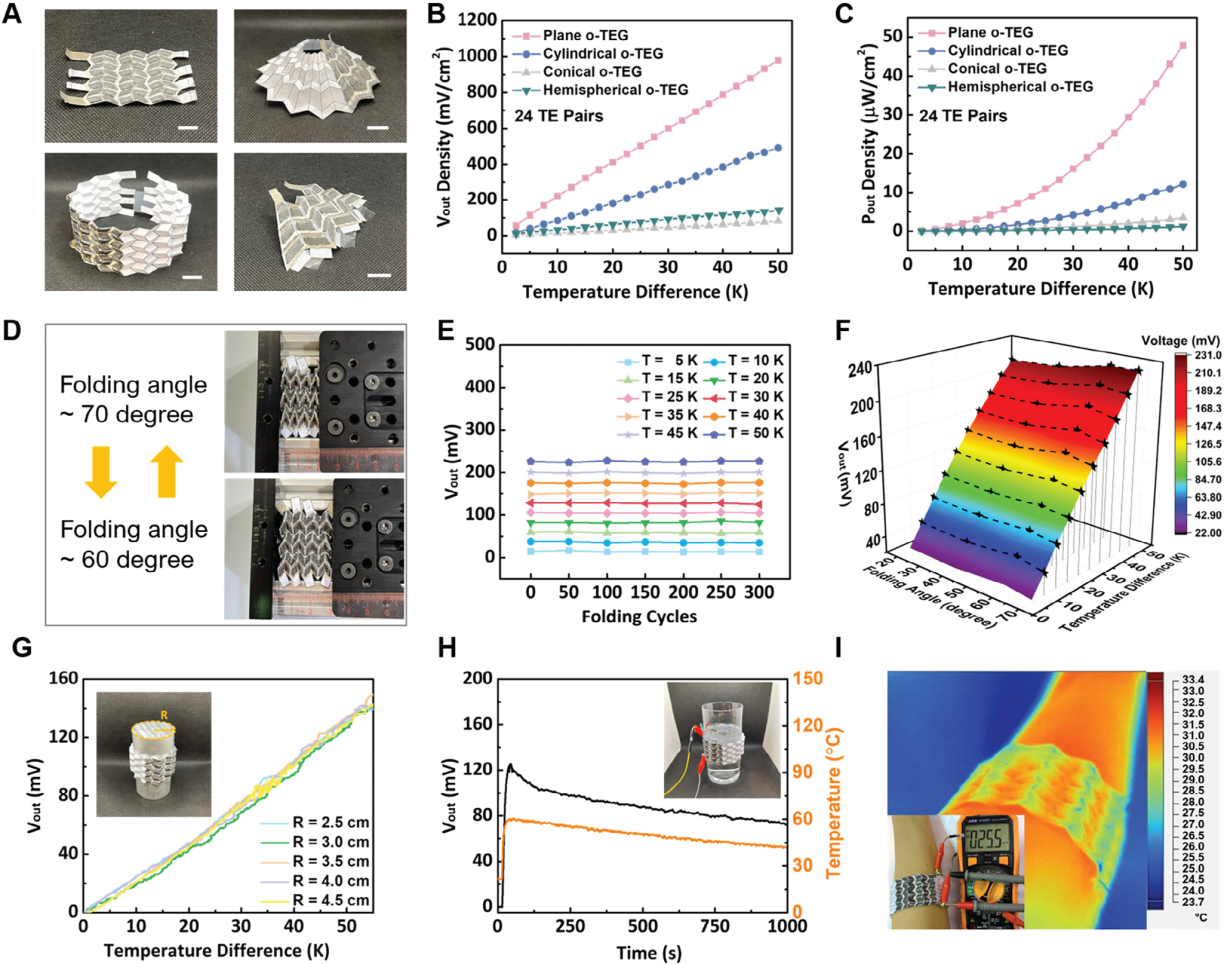
Characterization of the o‐TEDs. A) Optical images for plane o‐TED, cylindrical o‐TED, hemispherical o‐TED, and conical o‐TED. (Scale bar is 10 mm.) B) *V*_out_; C) the maximum *P*_out_ for plane o‐TED, cylindrical o‐TED, hemispherical o‐TED, and conical o‐TED. D) Schematic diagrams of plane o‐TED under different folding angles during the cycling tests. E) Cycling folding tests for the plane o‐TED under 0 to 300 folding cycles with 50‐cycle intervals in between. F) *V*_out_ for plane o‐TED under the temperature difference from 0 to 50 °C in different folding angles (20, 40, 60, and 70°). G) *V*_out_ measures cylindrical o‐TED with different targeting cylinder radii (2.5–4.5 cm, with 0.5 cm interval in between). Energy harvesting: H) a glass of hot water; I) a human arm by the cylindrical o‐TED.

Based on the origami designs, four types of o‐TEDs targeting multiple surfaces, respectively, are demonstrated in **Figure** **4**A. The cross‐section view of the region where the TE segment lies is shown in Figure S8A (Supporting Information) with the four distinct layers. The enlarged image of Figure S8B (Supporting Information) clearly shows the TE particles inside. As the TE particles were prepared by grinding and sieving with a mesh size of 200, the particle size was well controlled below 74 µm. The output performance for these four types of o‐TEDs with 24 TE pairs were provided in Figure 4B,C. With the entire bottom zigzag lines contacted with the heat source, the plane o‐TED achieved the highest *V*_out_ density of 0.98 V cm^−2^ (978.91 mV cm^−2^) when Δ*T* reached 50 K, then followed by the cylindrical o‐TED, hemispherical o‐TED, and conical o‐TED with the maximum *V*_out_ density of 0.49 V cm^−2^ (492.24 mV cm^−2^), 0.08 V cm^−2^ (81.65 mV cm^−2^), and 0.14 V cm^−2^ (141.52 mV cm^−2^), respectively. The maximum *P*_out_ was tested by paralleled connecting a resistance with the value equal to the device's inner resistance. The maximum *P*_out_ density can reach 47.87, 12.22,  3.49,  and 1.23 µW cm^−2^ for plane, cylindrical, conical, and hemispherical types, respectively. The comparison of our o‐TED with previously reported flexible (or foldable) TEDs, origami (or kirigami) TEDs, and wearable on‐body TEDs, was summarized in Tables S1 and S2 (Supporting Information), and Figure S13 (Supporting Information), respectively, demonstrates a first‐class output performance,^[^ ^7^, ^9^, ^10^, ^22^, ^23^, ^24^, ^25^, ^26^, ^27^, ^28^, ^29^, ^30^, ^31^, ^32^, ^33^, ^34^, ^35^, ^36^, ^37^, ^38^, ^39^, ^40^, ^41^ ^]^ especially compared to the TE ink‐based TEDs. TEDs with hot‐sintered TE cuboids were also included in the comparison table. Although they have demonstrated higher power density, the *V*_out_ density is low, which further demonstrates the superiority of the origami design in achieving large *V*_out_.

As plane o‐TED and cylindrical o‐TED could be folded with different folding angles, the stability of folding these two types of o‐TEDs was further verified. As illustrated in Figure 4D, the plane o‐TED with 24 TE pairs was circularly folded from its original state (60° folding angle) to its ultimate folding state (70° folding angle), and the *V*_out_–Δ*T* relation was tested every 50 folding cycles. Compared with *V*_out_ under different Δ*T* under every 50 folding cycles (Figure 4E), the o‐TED demonstrates good stability and repeatability in its output performance. The *V*_out_–Δ*T* relations for o‐TED were also tested under different folding angles (20°, 40°, 60°, and 70°). When the o‐TED is folded to its ultimate 70°, the *V*_out_ shows a slight increase by 6.37% (Δ*T* = 50 K) compared to that of o‐TED at the folding angle of 20°. The *V*_out_–Δ*T* relation for cylindrical o‐TED on metal rods with different bending radii (from 2.5 to 4.5 cm, with a 0.5 cm interval in between) was collected in Figure 4F, with a relatively stable output performance. The energy harvesting performance for the cylindrical o‐TED was verified by wrapping the o‐TED around a glass bottle and a human arm. As shown in Figure 4H,I the device *V*_out_ could reach 125.35 and 25.50 mV when it absorbed heat from the hot glass of water (Δ*T* = 39 K) and body heat (Δ*T* = 8 K), respectively.

## 5 Thermal Panel Application of o‐TEGs

**Figure 5:**
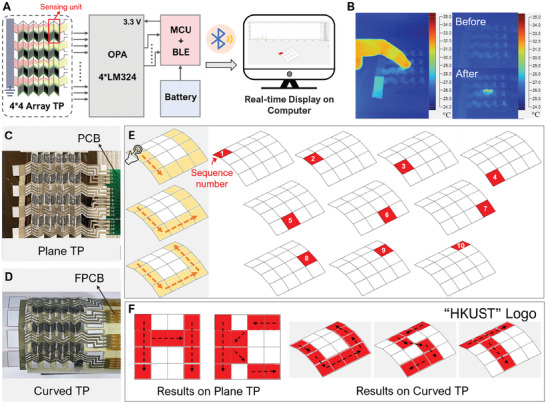
Plane and curved thermal touch panel (TTP) based on o‐TEDs. A) Schematic diagram of circuit design for a four‐by‐four TTP. B) Infrared images before and after the finger touch. Photo images: C) plane TTP based on the plane o‐TED; D) curved TTP based on the cylindrical o‐TED. E) The real‐time perception of the finger touch on a plane TTP with a four‐by‐four pixel. The right figures demonstrate the touching sequence when writing the letter “U”. F) Demonstration of the “HKUST” Logo using both plane and curved TTPs.

The 3D origami design of o‐TED achieves high *V*_out_ per TE pair than the traditionally used “π” structure, making it possible and more competitive when used as an individually controlled sensing pixel in a large array panel for thermal sensing. The origami circuit design of the thermal touch panel (TTP) is shown in **Figure** **5**A. Every basic folding element with four quadrangles contains one TE pair with N‐ and P‐type TE segments on one side while leaving the two other quadrangles for circuit design. The TE segments were connected and individually controlled by the designing circuit. Altogether, 16 *V*_out_ signals, representing 4 × 4 pixels in the sensing array, were collected and further transmitted to the computer for temperature matric display via. Bluetooth (ESP32). A thin layer of Parylene‐C (3 µm) electrically insulated the whole device. As shown in Figure 5B, the finger touch can naturally construct a Δ*T* of 10 K on the two sides of the TE pair, which allows us to use this 4 × 4 array as the handwriting TTP. Based on different origami designs, we fabricated plane and curved TPs targeting plane and cylindrical surfaces, respectively (Figure 5C,D). To better fit the cylindrical surface, the flexible PCB was used to connect the curved TTP with the data acquisition and transmit system, and detailed information for the data collection system was provided in Figures S10 and S12 (Supporting Information). It was worth mentioning that Bluetooth (ESP32) was used to achieve real‐time signal transmission, which enlarged its working scenario to remote information transmission. In Figure 5E, we have demonstrated the letter “U” on the computer display and the number inside each figure representing the handwriting sequence on TTP. Then, in Figure 5F, we wrote the letters “H” and “K” on the plane TTP and “U,” “S,” and “T” on the curved one. Altogether, we successfully demonstrated the “HKUST” Logo. The testing movies for the handwriting TTP are provided in the Movies S1–S5 (Supporting Information) showing a relatively fast response when the finger touches the TTP. Tests on the sensing performance of TTP were included in Figure S10 (Supporting Information), showing a fast response (≈2.34 s) with relatively good sensing linearity (coefficient of determination (*R* ^2^ = 0.99908)). Other experiments, including infrared light sensing, were demonstrated in Figure S10 (Supporting Information), which has expanded its application area to the photodetector for localized heat detection.

## 6 Conclusion

To conclude, we have demonstrated the programmable designed, target surface conformable paper‐based TE devices with significantly enhanced output performance. These innovative origami crease designs make it possible to conduct all fabrication processes on 2D papers while folding the TE device to fit the 3D structured surfaces. By changing the heat transferring direction within the screen‐printed TE laminates, a huge enhancement has been made to the output performance. Comparisons have been made between the o‐TEDs with the “π” structure one via. both the experiment tests and simulation, verifying the large performance improvement in both *V*_out_ and energy conversion efficiency. Based on the origami structure, the o‐TEDs consisting of 24 TE pairs have been fabricated, which can generate up to 0.98 V cm^−2^ and a power output of 47.87 µW cm^−2^ at Δ*T* of 50 K. Moreover, the plane and cylindrical o‐TEDs are further developed for fast and linear response plane and curve TTP, opening more application aspects for TEDs.

## Experimental Section

### Synthesis of the N‐Type and P‐Type TE Inks

N‐type Bi_2_Te_2.7_Se_0.3_ and P‐type Bi_0.5_Sb_1.5_Te_3_ ingots were purchased from Wuhan SAGREON Co., Ltd. The ingots were manually grinded and sieved with the mesh size of 74 µm to produce the TE powder. To prepare the binder solvent, ethyl alcohol, DI water, and methyl cellulose (Aladdin, M112867, 1500 mPa s) were mixed in the weight ratio of 25:25:1 and then stirred for 2 h to produce a clear solvent without bubbles and powders inside. After that, the N‐type and P‐type powders were added to the binder solvent with the mass concentration of 86.67 and 84.62 wt.%, respectively, and stirred for 3 h to get evenly dispersed and viscous TE inks.

### Fabrication of o‐TEDs

The pattern sizes for Ag paste electrodes, TE segments, and conductive cloth tapes used for reinforcing the electrical connection on the crease regions were pre‐designed via. AutoCAD based on the programmable method in the Supporting Information. Ag electrodes were screen printed on the paper substrate via. patterned PET thermal released film mask (thickness ≈100 µm), while the TE segments were screen printed by patterned PVC masks (thickness ≈500 µm). Conductive cloth tape was first patterned by the laser cut process (laser power of 7.6 W (±0.1 W) and a speed of 10 mm s^−1^) and then placed on the target region via. a pick‐and‐place method in the previously reported research.^[^ ^3^ ^]^ After that, the light‐cured epoxy resin was scraped on top of the TE segments via. the PVC masks (thickness ≈500 µm) and then cured under the UV light. Parylene‐C (≈4 µm) layer was deposited on the surface of o‐TED for electrical insulation. The HCM was prepared by mixing the AlN powder with Ecoflex in a proportion of 50 wt.% via. Kurabo PDMS Mixer/Deaerator (PHT‐MX1). It was then deposited on the bottom zigzag region by pre‐designed 3D printing contour with the extruder (Model: Nexus 6000 from Chemyx Inc.) in an extrusion speed of 10 mL h^−1^ (20 mL Syringe).

### Materials Characterization and Device Performance Measurement

SEM was used to characterize the surface morphology of the cross‐section view of the o‐TED and the TE segments. The Seebeck coefficient and electrical resistivity were measured by a simultaneous measurement system (CTA‐3). The thermal conductivity by the equation *κ* = *λρC*_P_ was evaluated, where *λ*, *ρ*, and *C*_P_ are the thermal diffusivity, material density, and specific heat capacity, respectively. The *λ* can be measured by a laser flash method (LFA‐457, Netzsch), while *ρ* and *C*_P_ were measured by the Archimedes method and a differential scanning calorimeter (TGA2/DSC3, Switzerland). The thermal conduct of the HCM was determined by the Hot disk (TPS1500). The *V*_out_ performance was measured on the constant temperature‐controlled hotplate (JF‐956A, SHUNSHENG Electronic Technology), and the data were collected using a data acquisition card of NI. All infrared images were captured by an infrared camera (Ti489 PRO FLUKE).

## Conflict of Interest

The authors declare that they have no conflict of interest.

## Supporting information

Supporting Information

Supplemental Movie 1

Supplemental Movie 2

Supplemental Movie 3

Supplemental Movie 4

Supplemental Movie 5

## Data Availability

The data that support the findings of this study are available in the supplementary material of this article.
