# Supplementary material for: Programmable and Surface‐Conformable Origami Design for Thermoelectric Devices
Source: Adv Sci (Weinh). 2024 Jan 2;11(10):2309052. doi: 10.1002/advs.202309052 (PMC10933682; doi:10.1002/advs.202309052)
Supplement: Supplementary file 1 — Supporting Information [file ADVS-11-2309052-s002.pdf]

ADVANCED SCIENCE
Open Access

Supporting Information

for *Adv. Sci.*, DOI 10.1002/advs.202309052

Programmable and Surface-Conformable Origami Design for Thermoelectric Devices

Yue Hou, Zhaoyu Li, Ziyu Wang, Xingzhong Zhang, Yang Li, Chang Li, Haizhong Guo* and Hongyu Yu**

Supporting Information

Programmable and Surface-conformable Origami Design for Thermoelectric Devices

Yue Hou, Zhaoyu Li, Ziyu Wang, Xingzhong Zhang, Yang Li, Chang Li, Haizhong Guo*, Hongyu Yu**

1) Programmable design for cylindrical origami

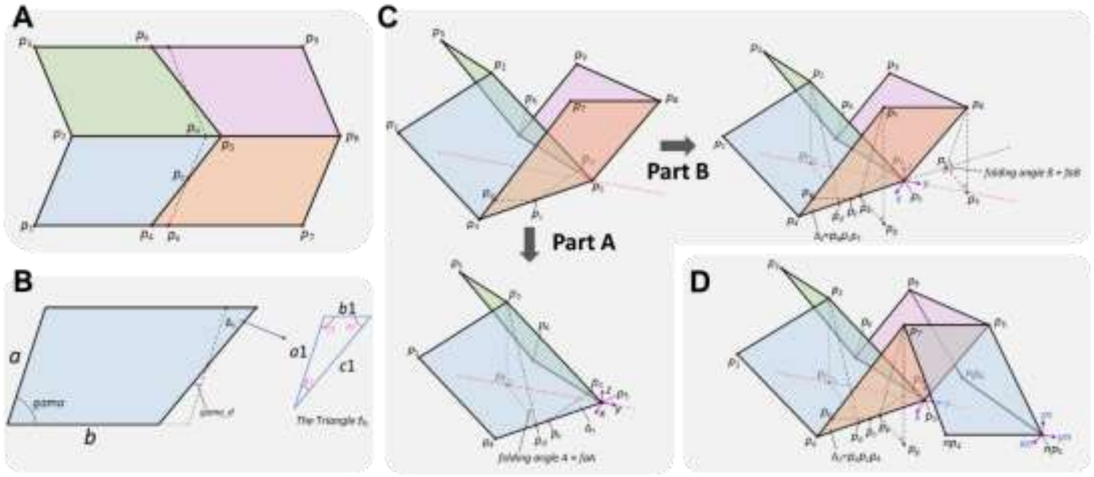

Figure S1. Programmable designs for cylindrical origami. (A) BFE in cylindrical origami. (B) Quadrangle in cylindrical origami. (C) Folded state of the cylindrical origami. (D) Propagation of cylindrical origami BFE.

The basic folding element (BFE) of the traditional Miura-ori structure that conforms to the planer surface comprises four parallelograms. By modifying the BFE of Miura-ori structure as in Figure S1A, a different folding result can be obtained, called cylindrical origami. As shown in Figure S1B, the parallelogram is an element in the BFE of Figure S1A, in which the parallelogram has three intrinsic parameters: shorter edge *a*, long edge *b,* and angle *gama*. Note that, such intrinsic parameters determine the shape of the whole origami and will not change in the folding process.

Adding an extra intrinsic parameter *gama_d* to the parallelogram, as shown in Figure S1B, a deformed quadrangle can be obtained, whose intrinsic parameters are shorter edge *a*, long edge *b*, angle *gama*, and angle *gama_d*. Then, for the triangle Δ_1 in Figure S1B, we can have the inner angles of Δ_1, as listed in Eq. (1).

$$\begin{aligned} \angle B1 &= gama\_d \\ \angle C1 &= \pi - gama\_d \\ \angle A1 &= gama - gama\_d \end{aligned} \tag{1}$$

Then, using the law of sines, we can have the edge length of Δ_1, as listed in Eq. (2).

$$\begin{aligned} a1 &= 0.5*a \\ b1 &= a1*sin(B1)/sin(A1) \\ c1 &= a1*sin(C1)/sin(A1) \end{aligned} \tag{2}$$

The folded cylindrical origami is shown in Figure S1C. Intuitively, the same as traditional origami. The cylinder origami is symmetric to its medial plane, i.e., the whole figure is symmetric to the plane $p_2p_5p_8$. For better analyzing the folding property, the folded cylindrical BFE is analyzed in two parts, part A of $p_1p_2p_3p_4p_5p_6$ and part B of $p_4p_5p_6p_7p_8p_9$.

- **Part A of BFE**

Part A is the left part of BFE in Figure S1C, which p1p2p3p4p5p6 forms. Point p_5 is taken as the origin to analyze the folding property, and the coordinate system associated with the origin (p_5) is shown in Figure S1C. Then, the whole folded BFE is symmetric to the plane *yoz*.

The angle formed by quadrangle $p_1p_2p_5p_4$ and plane *xoy*, e.g., *faA* in Figure S1C, is the folding angle of BFE. With the change of folding angle *faA*, the geometry of BFE is changing. E.g., the spatial location of $p_1p_2p_3p_4p_6$ is changing with folding angle *faA*. Then derivation process will be $\Delta p_2p_5p_d \rightarrow \Delta p_2p_dp_f \rightarrow \Delta p_5p_dp_f$.

In $\Delta p_2p_5p_d$, we have:

$$\overline{p_2p_5} = b + b1, \text{ (b1 is from Eq.(2))}$$

$$\overline{p_2p_d} = \overline{p_2p_5} * sin(\text{A1}), \text{ (A1 is from Eq.(1))} \quad (3)$$

$$\overline{p_5p_d} = \overline{p_2p_5} * cos(\text{A1})$$

In $\Delta p_2p_dp_f$, we have:

$$\overline{p_2p_f} = \overline{p_2p_d} * sin(faA), \text{ (} \overline{p_2p_d} \text{ is from Eq.(3))}$$

$$\overline{p_fp_d} = \overline{p_2p_d} * cos(faA), \quad (4)$$

In $\Delta p_5p_dp_f$, we have:

$$\overline{p_5p_f} = \sqrt{(\overline{p_5p_d})^2 + (\overline{p_dp_f})^2}$$

$$sin(\angle p_dp_5p_f) = \overline{p_dp_f} \;/\; \overline{p_5p_f} \quad (5)$$

Hence, from the shape of the above three triangles, the following geometry information can be obtained:

two points: p_2, p_c

$$p_2 = [0, -\overline{p_5p_f}, \overline{p_2p_f}]$$

$$p_c = [\overline{p_5p_c} * sin(\angle p_dp_5p_f), -\overline{p_5p_c} * cos(\angle p_dp_5p_f), 0] \quad (6)$$

four vectors: $\overrightarrow{p_5p_2}$, $\overrightarrow{p_5p_a}$, $\overrightarrow{p_ap_c}$, $\overrightarrow{p_5p_c}$

$$\overrightarrow{p_5p_2} = p_2 - p_5$$

$$\overrightarrow{p_5p_a} = b1 * (\overrightarrow{p_5p_2} / |\overrightarrow{p_5p_2}|)$$

$$\overrightarrow{p_5p_c} = p_c - p_5$$

$$\overrightarrow{p_ap_c} = \overrightarrow{p_5p_c} - \overrightarrow{p_5p_a}$$

Then, all points can be obtained:

$$p_1 = p_5 + \overrightarrow{p_5p_2} + 2*\overrightarrow{p_ap_c}$$

$$p_4 = p_5 + 2*\overrightarrow{p_5p_c}$$

$$p_3 = [-(p_1)_x, (p_1)_y, (p_1)_z] \quad (7)$$

$$p_6 = [-(p_4)_x, (p_4)_y, (p_4)_z]$$

- **Part B of BFE**

The spatial location of all quadrangle vertex points, e.g., $p_1p_2p_3p_4p_5p_6$, are obtained. Then, based on the same coordinate system that is associated at p_5, and with the shape of Part A, the folding geometry shape of Part B ($p_4p_5p_6p_7p_8p_9$) can be found. From the shape of Part A, we can find some geometry constraints to calculate the shape of Part B in BFE.

Since the BFE is symmetrical geometry, point p_8 falls on the plane *yoz*, and its coordinates can be supposed as $p_8 = [0, (p_8)_y, (p_8)_z]$. Then the following equations can be obtained.

$$|\overrightarrow{p_5p_8}| = b - b1$$

$$\mathrm{dot}(\overrightarrow{p_5p_8}, \overrightarrow{p_5p_4}) = |\overrightarrow{p_5p_8}||\overrightarrow{p_5p_4}|cos(\pi\text{-A1}) = -(b\text{-}b1)(2*c1)cos(\text{A1}) \quad (8)$$

where $b1$, $c1$ and A1 are from Eq. (1) and Eq. (2).

With two equations in Eq. (8), the coordinates of p_8 can be solved. From Figure S1A, we can have $\overrightarrow{p_5p_8}$ // $\overrightarrow{p_4p_7}$. Hence, the point p_7 can be calculated as below.

$$p_7 = p_4 + (|\overrightarrow{p_4p_7}|)*(\overrightarrow{p_5p_8}\ /\ |\overrightarrow{p_5p_8}|) = p_4 + (b + b1)*(\overrightarrow{p_5p_8}\ /\ |\overrightarrow{p_5p_8}|) \quad (9)$$

The final point p_9 can then be calculated as

$$p_9 = [-(p_7)_x, (p_7)_y, (p_7)_z] \quad (10)$$

The angle formed by plane $p_4p_5p_8p_7$ and plane *xoy*, is the resultant folding angle at the side of Part B, i.e., the *faB* in Fig. S1C. The *faB* can be calculated as below.

$$sin(faB) = |p_8p_h| / |p_8p_i| = (p_8)_z / [(b - b1)*sin(\mathrm{A1})] \quad (11)$$

By now, all points of BFE ($p_1p_2p_3p_4p_5p_6p_7p_8p_9$) are calculated and the shape of cylindrical origami BFE can be fully determined. Next, how the BFE is propagated to form the array of cylindrical origami will be introduced.

- **Propagation of cylindrical origami BFE**

The folded origami geometry will remain a planar shape in traditional origami, where the BFE consists of four parallelograms. However, with the added intrinsic parameter *gama_d*, the folded origami is no longer in a planar shape, and the BFE array will be cylindrical.

Based on one BFE, the following BFEs can be propagated. Figure S1D shows the current BFE with the Part A of the next BFE. The points of the next BFE are $np_1np_2np_3np_4np_5np_6np_7np_8np_9$. The coordinate system (*xnynzn*) associated with point np_5, is the coordinate system to indicate the folding process of the next BFE.

Now, we need to figure out the spatial relation between *xyz* and *xnynzn*, and how the folding process is propagated.

Since the BFE is symmetrical geometry, we can assume that the coordinate of np_5 in xyz is $np_5 = [0, (np_5)_y, (np_5)_z]$. Hence, the following equations can be obtained.

$$|\overrightarrow{p_8np_5}| = b + b1$$
$$\mathrm{dot}(\overrightarrow{p_8np_5}, \overrightarrow{p_8p_7}) = |\overrightarrow{p_8np_5}||\overrightarrow{p_8p_7}|cos(\pi\text{-A1}) = -(b+b1)(a)cos(\text{A1}) \quad (12)$$

With two equations in Eq. (12), the two unknowns $(np_5)_y$ and $(np_5)_z$ can be solved, thus, the coordinate of np_5 in xyz is obtained.

$$np_4 = p_7 + (b - b1)*(\overrightarrow{p_8np_5} / |\overrightarrow{p_8np_5}|)$$
$$np_6 = [-(np_4)_x, (np_4)_y, (np_4)_z] \quad (13)$$

Hereafter, we can obtain that:

$$np_5 = p_5 + \overrightarrow{p_5p_8} + \overrightarrow{p_8np_5}$$
$$zn = \mathrm{cross}\,(\overrightarrow{np_5np_6}, \overrightarrow{np_5np_4}) \quad (14)$$

From Eq. (14), the transformation from xyz to $xnynzn$ can be obtained.

2) Programmable design for quadrics surface conformed origami

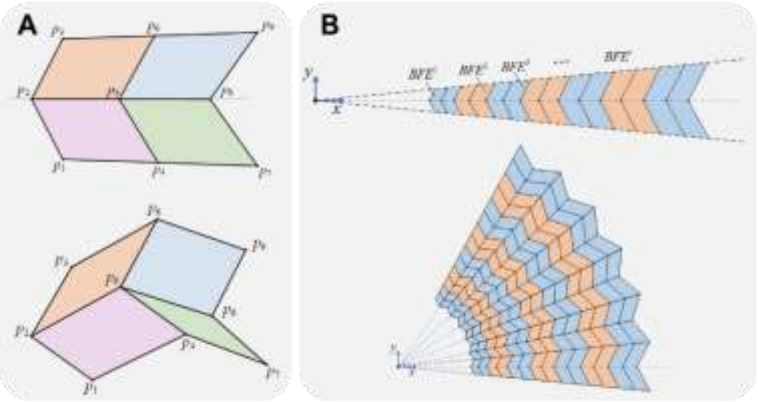

Figure S2. (A) BFE in cylindrical origami. (B) One column and a circular array of BFEs.

We refer to the method of Axisymmetric Origami Design proposed by Hu et al.[1] for designing the origami structure to cover the axisymmetric surface, in which the designed origami structure will also be axisymmetric. As shown in **Figure S2A**, the basic folding element (BFE) is composed of nine points and symmetrically to the line of p2-p5-p8. Before generating the entire origami structure, one column of origami BFE will be designed first to cover the section profile of the axisymmetric surface, as shown in Figure S2B. Note that, in the folding process, a column of origami BFE will always be symmetrical to its symmetry plane in any folding status of the origami structure. Then, by circular arraying such a column of origami BFE, we can obtain an origami structure to cover the axial symmetry surface, as shown in Figure S2B.

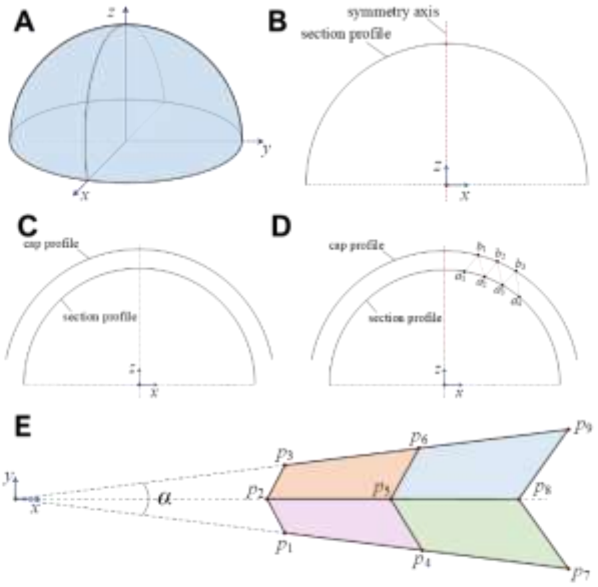

Figure S3. (A) axisymmetric surface to be covered. (B) section profile. (C) the cap profile above the section profile. (D) control points for determining the BFE. (E) Included angle α of the BFEs.

We now introduce how to design one column of BFE. An example axisymmetric surface is shown **Figure S3A** in which the section profile of the surface will be used to design the column of the BFE, as shown in Figure S3B. The section profile of the surface (Figure S3B) is used to restrict the lower boundary of the BFEs, while another *cap profile* restricts the upper boundary of the BFEs, as shown in Figure S3C. The designed one column of the BFE structure will then be sandwiched by the section profile and the cap profile.

The one column of BFE is marked as $\Psi=\{BFE^1, BFE^2, BFE^3, \ldots\}$, as shown in Figure S2B. Since the BFEs structure has only one degree of freedom in the folding process, the folding status of Ψ can be represented as the function of folding angle θ, i.e., Ψ(θ) $\theta \in [0, 180°]$. The design of the BFE structure is then under a given folding angle θ. In other words, for a specific folding status, we design the BFE structure to fit the surface

section profile.

The section profile and the cap profile are used to design the column BFEs. The user can select the cap profile. For the case in Figure S3A., which is a sphere surface, the cap profile (Figure S3C) is selected as the offset curve of the section profile. Then, two series of sample points will be selected on the section profile and the cap profile, respectively, i.e., $\{a_1, a_2, a_3, \ldots\}$ from the section profile and $\{b_1, b_2, b_3, \ldots\}$ from the cap profile, as shown in Figure S3D. $\{a_1, a_2, a_3, \ldots\}$ and $\{b_1, b_2, b_3, \ldots\}$ will then be the *control points* for designing the origami structure. While for designing the axisymmetric origami structure to cover the axisymmetric surface, the selected sample points should satisfy the geometry condition that $k(a_i, b_i) = - k(a_{i+1}, b_i)$, wherein $k(a, b)$ indicates the slope of the line that goes through point a and b.

Another parameter to determine is the spanned angle of BFE, as the angle α shown in Figure S3E. The spanned angle α of BFE changes with the folding of the origami structure, and we need to assign an angle α for $\Psi(\theta)$. Note that all BFEs in Ψ always have the same spanned angle, as shown in Figure S2B.

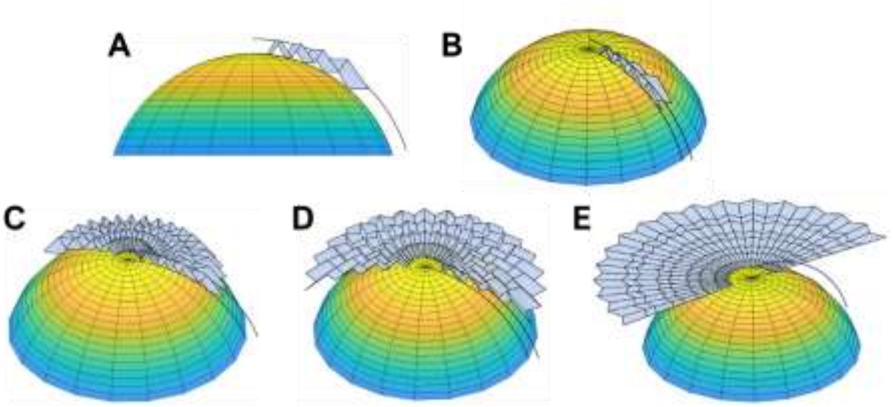

Figure S4. (A) front view and (B) oblique view of one column of BFE. (C) Designed origami structure for fitting the axisymmetric surface. Designed origami structure

folded with the folding angle being (D) 120°and (E) 180°.

With the above specifications, the structure of BFE can now be determined. For the first BFE, i.e., BFE^1, three sample points a_1, b_1, and a_2 are, respectively, the structure points p_2, p_5, and p_8, with the folding angle being θ, recalling Figure S2A. Hereafter, with the assigned spanned angle α, other structure points p_1, p_4, p_7, p_3, p_6, and p_9 can be calculated accordingly.

Then for the second one, i.e., BFE^2, sample points a_2, b_2, and a_3 will be the structure points p_2, p_5, and p_8 of BFE^2. Note that, the structure points p_1, p_2, and p_3 of BFE^2, are exactly the structure points p_7, p_8, and p_9 of BFE^1. Thus, only the structure points p_4, p_6, p_7, and p_9 of BFE^2 need to be determined, which can be similarly calculated following the design procedure of BFE^1.

Therefore, step by step, one column of BFE can be obtained, which is sandwiched by the section profile and the cap profile when the folding angle is θ. **Figure S4A** and B show the example of designing one column of the BFE, wherein the element number is 4, and the folding angle is 60°. By duplicating the column of BFE in a circular pattern, we can obtain an origami structure to fit the surface, as shown in Figure S4C. We also display its other folding status for the designed origami structure, i.e., folding angle 120° (Figure S4D and 180° (Figure S4E).

Based on the aforementioned origami design, other quadrics surface conformable designs, including hemisphere, conical, paraboloid, and ellipsoid surfaces with specific parameters, are listed in Figure S5.

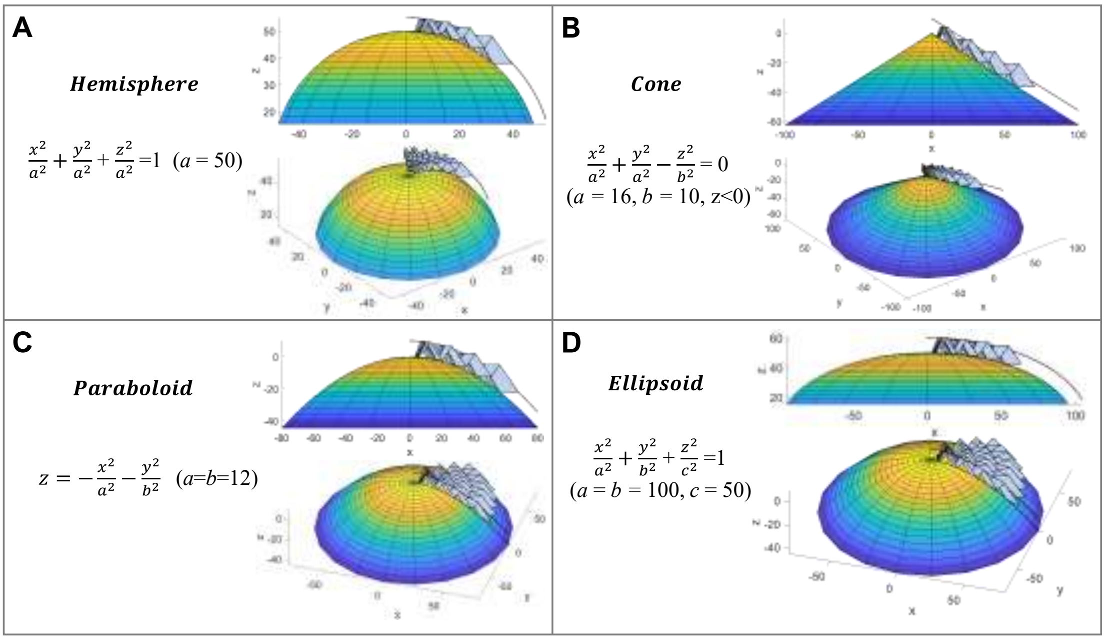

Figure S5. Programmable designs for origami substrates conformable to (A) hemisphere, (B) Conical, (C) Parabola, and (D), Ellipsoid surfaces.

3) Experiment details

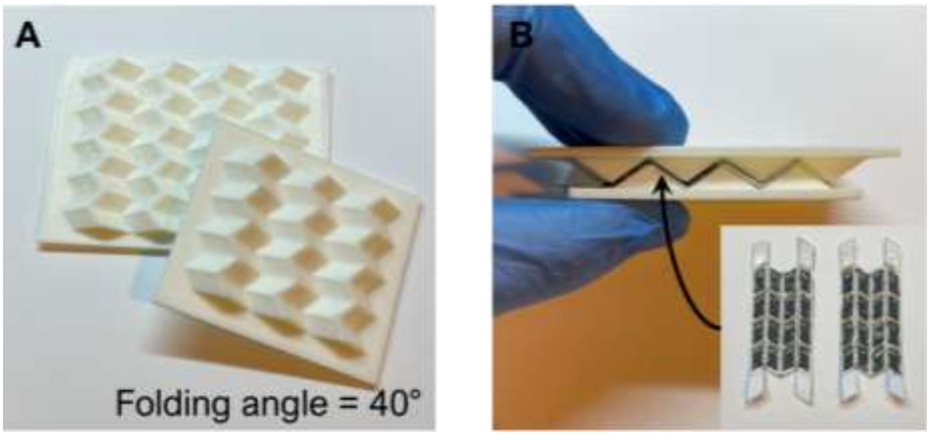

Figure S6. 3D printing molds which help facilitate the folding of o-TEGs. (A) A pair of 3D printing molds targeting the folding state of 40 degrees. (B) The folding process of the o-TEG.

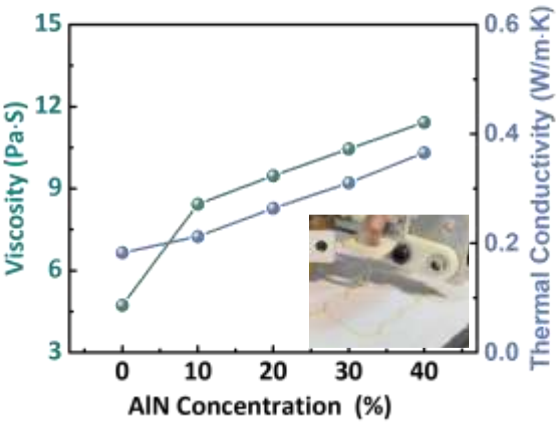

Figure S7. Viscosity and thermal conductivity of HCM under different AlN concentrations.

3) Materials characterization

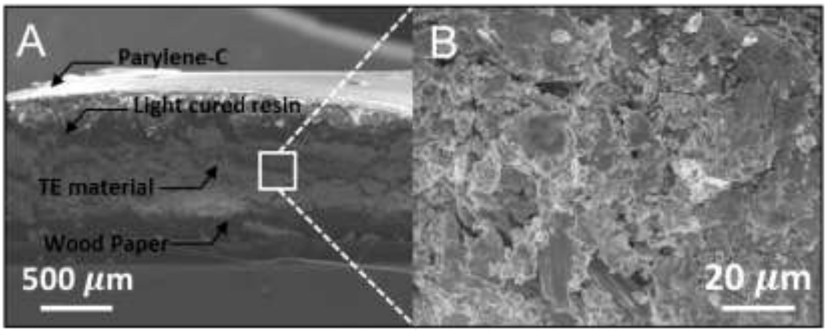

Figure S8. (A) The cross-section view of the TE segment of o-TEG. (B) Enlarged image of the TE material.

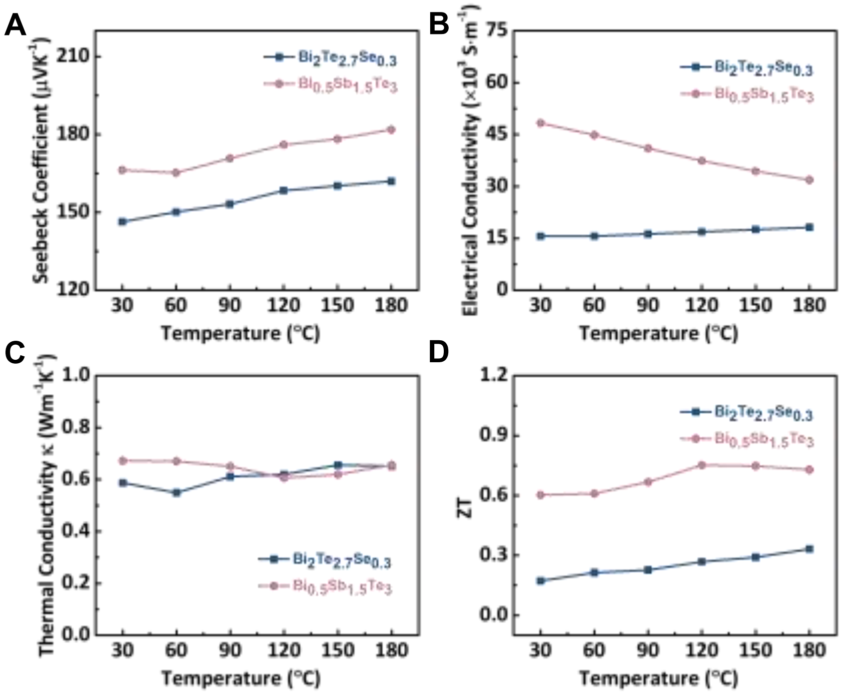

Figure S9. Thermoelectric properties of the TE ink. (A) Seebeck coefficient (*S*), (B) Electrical conductivity (ρ), (C) Thermal conductivity(κ), and (D) ZT value of the N-type ($Bi_2Te_{2.7}Se_{0.3}$) and P-type ($Bi_{0.5}Sb_{1.5}Te_3$) TE material under the temperature of 30 °C to 180 °C, with 30 °C intervals in between.

The material properties of the two screen-printed TE inks are summarized in **Figure S9** with the maximum ZT value of 0.75 and 0.33 for n-type $Bi_2Te_{2.7}Se_{0.3}$ and p-type $Bi_{0.5}Sb_{1.5}Te_3$ inks, respectively. All trends of *S*, ρ, and κ values under the testing temperature are consistent with the TE material characterization in the previous reports[2,3].

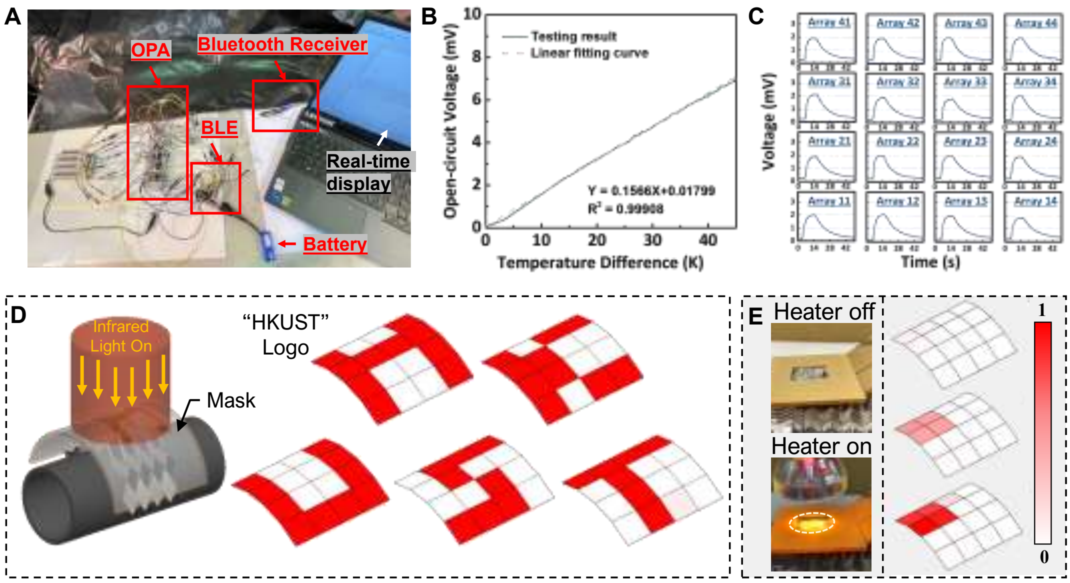

Figure S10. (A) Testing scene of the handwriting TTP. (B) The V_{out}-ΔT relations for one single pixel of the plane TP. (C) Hand touch testing for all sixteen pixels on plane TP. (D) Working principle of thermal curve panel for information transmission and the "HKUST" logo demonstration based on the thermal curve panel. (E) Localized infrared light heating and the results are shown on the computer.

Figure S10A shows the testing scene of the plane TTP. Four operational amplifiers (OPA) are connected to the TTP to amplify the V_{oc} by 200 times. A pair of ESP-32, one as a microcontroller (MCU) with Bluetooth function, and the other works as the Bluetooth receiver connected to the computer. The 4 by 4 real-time display corresponding to the TTP pixel is achieved through MATLAB. The V_{out} under the temperature difference was collected in Fig. S6B with excellent linearity (coefficient of determination ($R^2 \approx 0.99908$)). All 16 pixels' performance tested by finger touch is summarized in Figure S10C, showing good consistency.

Another device application is the infrared light image display. Here, thick paper masks with "H", "K", "U", "S", and "T" holes that could block part of the light were prepared and packaged around the curve TTP. When the infrared light is on, the corresponding letters can be shown on the remote display (Figure S6D and Figure S6E).

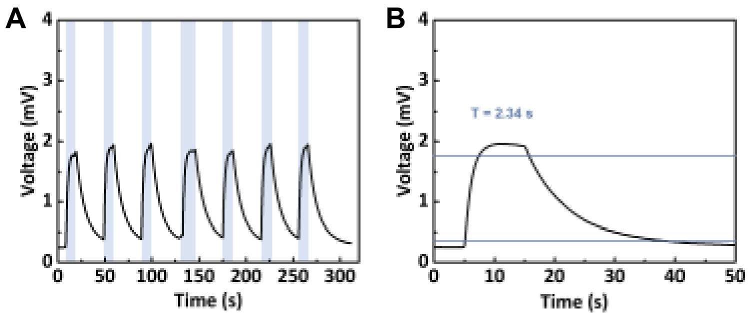

Figure S11. (A) Cycling finger touching test on one single sensing pixel. (B) Response time of TP when the finger touches the sensing unit.

The cycling finger-touching test is demonstrated in Figure S11A, showing good stability. In Figure S11B, the response time of 2.34s is calculated.

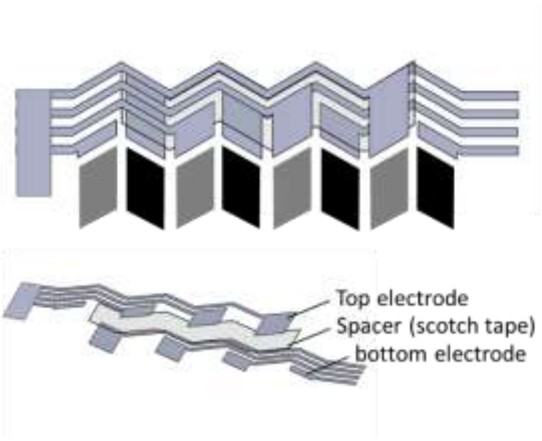

Figure S12. Electrical design for one array of TTP.

During the TTP fabrication, two methods have been used to avoid short circuit issue. First, as shown in Figure S12, one patterned layer of scotch tape was used as the spacer to separate two layers of electrode. Second, a layer of Parylene-C (~5μm) was deposited on top of device, which could avoid the short circuit issue when human finger touches the sensing pixel.

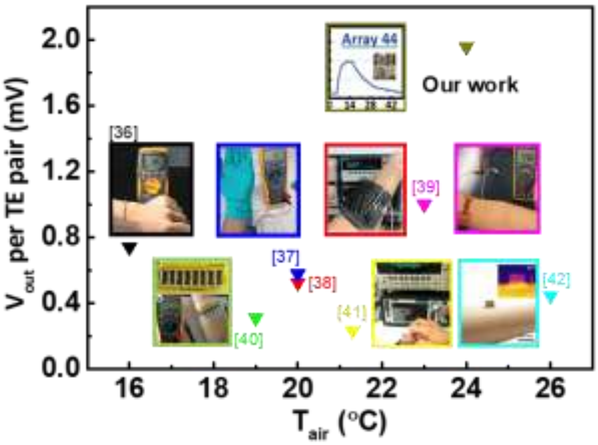

Figure S13. The output performance of o-TEG in this work compared with several literature data.

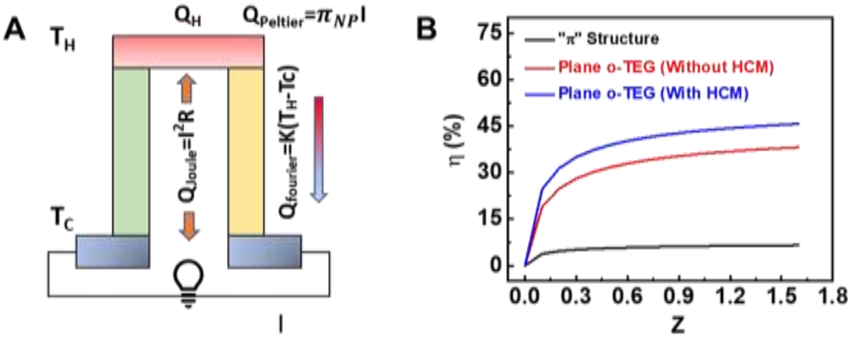

Figure S14. (A) The energy conservation inside a single pair of TE when the system is steady. (B) The relationship between η_{max} and the Z for three structures with different T_H and T_C.

When the system is in a steady state, with fixed temperatures at the hot and cold ends are T_H and T_C, respectively. The external environment inputs energy from the hot end to maintain the temperature at the hot end, and all the input energy is absorbed by the device at the hot end. To establish a thermal balance equation at the hot end, the received heat consists of the external input heat Q_H and half of the Joule heat, which is $0.5I^2R$. As shown in Fig. S14A, the heat transferred downwards includes the Peltier heat πI and the Fourier heat $K\Delta T$. Here, R represents the total resistance of the thermoelectric leg, I represents the current generated by the Seebeck voltage in the circuit, and K represents the total thermal conductivity of the thermoelectric leg, which signifies the heat flow through the unit temperature difference. The equation expression is as follows:

$$Q_{Peltier} + Q_{Fourier} = \frac{1}{2} Q_{Joule} + Q_H$$

$$\pi_{PN} I + K(T_H - T_C) = \frac{1}{2} I^2 R + Q_H$$

When the load resistance is R_L, the current and output power and be expressed as:

$$\mathrm{I} = \frac{S_{PN}(T_H - T_C)}{R + R_L}$$

$$\mathrm{P} = I^2 R_L$$

As the heat transferring coefficient η can be defined as the ratio of output power and input heat. Here, the Peltier coefficient can be replaced by the Seebeck coefficient ($\pi = S * T_H$) using the Kelvin relationship. Then the η can be expressed as:

$$\eta = \frac{P}{Q_H} = \frac{I^2 R_L}{\pi_{PN} I + K(T_H - T_C) - \frac{1}{2} I^2 R}$$

$$= \frac{{S_{PN}}^2 (T_H - T_C)^2 R_L / (R + R_L)^2}{\frac{S_{PN}^2 T_H (T_H - T_C)}{R + R_L} + K(T_H - T_C) - \frac{1}{2} {S_{PN}}^2 (T_H - T_C)^2 R / (R + R_L)^2}$$

$$= \frac{{S_{PN}}^2 (T_H - T_C)^2 R_L}{S_{PN}^2 T_H (R + R_L) + K(R + R_L)^2 - \frac{1}{2} {S_{PN}}^2 (T_H - T_C)^2 R}$$

$$= \frac{{S_{PN}}^2 (T_H - T_C) R_L}{\frac{1}{2} {S_{PN}}^2 (T_H - T_C)^2 R + K(R + R_L)^2 + S_{PN}^2 T_H R_L}$$

$$= \frac{{S_{PN}}^2 (T_H - T_C)}{\left[\frac{1}{2} {S_{PN}}^2 (T_H - T_C)^2 R + K R^2\right] \frac{1}{R_L} + K R_L + S_{PN}^2 T_H + 2KR}$$

When $R_L = \sqrt{\frac{\frac{1}{2} {S_{PN}}^2 (T_H + T_C)^2 R + K R^2}{K}}$, η can obtain its maximum value.

For the thermoelectric device, $\bar{T} = \frac{T_H + T_C}{2}$, $\mathrm{Z} = \frac{S_{PN}^2}{RK}$, where the total resistance and total thermal conductivity of a pair of thermoelectric arms are used to define the thermoelectric value Z of the device. Therefore, capital letter $Z\bar{T}$ is directly point to thermoelectric device, which can be expressed as:

$$\mathrm{Z}\bar{T} = \frac{S_{PN}^2}{RK} \frac{T_H + T_C}{2}$$

When the direction of the heat flow current is the same, the ratio of the material's conductivity to thermal conductivity (or the ratio of thermal resistance to resistance) is equal to the ratio of electrical conductivity to thermal conductivity. At the same time, considering the Seebeck coefficient, electrical conductivity, thermal conductivity is temperature dependent, so it is used to use lowercase z times T as a dimensionless

thermoelectric value to describe the thermoelectric performance of the material at a certain temperature. So the lowercase zT can be expressed as:

$$\mathrm{zT} = \frac{S^2\sigma}{\kappa}T$$

As $\mathrm{Z} = \frac{S^2}{RK}$, then

$$\eta_{max} = \frac{T_H - T_C}{T_H}\frac{\sqrt{Z\bar{T}+1}-1}{\sqrt{Z\bar{T}+1}+T_C/T_H}$$

Then the relationship between η_{max} and Z can be depicted in Fig. 3B.

And Z can be further elaborated as

$$\mathrm{Z} = \frac{S^2}{RK} = \frac{(S_P - S_N)^2}{(\frac{l_P}{A_P}\rho_P + \frac{l_N}{A_N}\rho_N)(\frac{A_P}{l_P}k_P + \frac{A_N}{l_N}k_N)}$$

As we can see from the above equation, the value of capital Z is related to the 1) Seebeck coefficient of the thermoelectric material, and 2) size of the TE Legs.

Therefore, for all three types of structures, capital Z value for all three types can be estimated by the given value S_P, S_N, k_P, k_N ρ_p and ρ_N all at the T of ~30 °C, and these values are summarized below.

	S_P (μV/K)	S_N (μV/K)	ρ_P (μOhm·m)	ρ_N (μOhm·m)	$\frac{l_P}{A_P}$ (m^{-1})	$\frac{l_N}{A_N}$ (m^{-1})	$\frac{A_P}{l_P}$ (m)	$\frac{A_N}{l_N}$ (m)	k_P (W/mK)	k_N (W/m K)	Z (K^{-1})
Π structure	164.1596	-145.0687	20.4990	70.7577	9.259	9.259	0.1080	0.1080	0.67	0.59	8.318E-4
Plane o-TEG	164.1596	-145.0687	20.4990	70.7577	3000	3000	0.0003	0.0003	0. 67	0.59	9.240E-4

As capital Z value for all three structure are at the same order of magnitudes, when taken the Z value back to the Fig. S14B, the η_{max} for all π structure, plane o-TEG

with and without HCM still demonstrated the same rising characteristic as the one we mentioned in the manuscript. When T is around 30 °C, the η_{max} are 0.12%, 0.43%, and 0.60%, respectively, and the same rising trend still demonstrated as the T grows, demonstrating the superiority of the origami structure design and add of the HCM materials.

Movie S1-S2. Demonstration for capital letters "H" and "K" on plane thermal panel (TP).

H_LOGO_On plane TP.mp4 K_LOGO_On plane TP.mp4

Movie S3-S5. Demonstration for capital letters "U", "S", and "T" on curved thermal panel (TP).

U_LOGO_On curved TP.mp4 S_LOGO_On curved TP.mp4 T_LOGO_On curved TP.mp4

Table S1. Comparison on the output performance of our o-TEG with previously reported flexible or foldable TEGs.

TE Materials (N/P)	Substrate	Flexible or foldable	ΔT (K)	Voltage Output Density per K ($mV/K\cdot cm^2$)	Power Density per K ($\mu W/K\cdot cm^2$)	Reference
**TE inks** ($Bi_2Te_{2.7}Se_{0.3}$/ $Bi_{0.5}Sb_{1.5}Te_3$)	Paper	Yes	50	19.58	0.96	Our Work
**TE inks** Bi2Te3/BC nanofiber	Paper	Yes	55	0.89	0.0075	[22]
**TE inks** Bi2Se0.3Te2.7 Bi0.5Sb1.5Te3	Paper	Yes	35	0.01	0.000015	[23]
**TE inks** Doped Bi2Te3 and Sb2Te3	Polyimide	Yes	40	18.58	0.00059	[24]
**TE inks** TiS_2	Polyethylene naphthalate	Yes	30	14.87	1.59	[25]
**TE inks** Ag_2Se/Cu_2Se	Kapton	Yes	45	19.28	0.51	[26]
**TE inks** PEDOT:Tos/CuI	/	Yes	20	0.17	0.0065	[11]
**TE inks** CNT	Polyurethane	Yes	30	0.52	0.04	[27]
**Deposited thin film** (Sb2Te3/Bi2Te3)	Polyimide	Yes	~36	2.40	0.091	[12]
**Deposited thin film** $Bi_2Te_{2.7}Se_{0.3}$ $Bi_{0.5}Sb_{1.5}Te_3$	Polyimide	Yes	95	10.53	0.20	[28]
**TE cuboids** (Sb_2Te_3/Bi_2Te_3)	Silicon Elastomer	Yes	18.9	5.82	7.28	[9]
**TE cuboids** ($Bi_2Te_{2.7}Se_{0.3}$ $Bi_{0.5}Sb_{1.5}Te_3$)	Aerogel/PDMS	Yes	28.5K / 11K	7.27	1.27	[29]

Table S2. Comparison of the materials and output performance of o-TEG with previously reported origami or kirigami-designed TEG.

TE Materials (N/P)	Origami/ Kirigami design	T_{hot}(°C)/ ΔT (K)	Voltage Output (mV)	Voltage Output Density (mV/cm^2)	Voltage Output Density per K ($mV/K\cdot cm^2$)	Power (μW)	Power Density ($\mu W/cm^2$)	/Power Density per K ($\mu W/K\cdot cm^2$)	Reference
PEDOT nanowires/TiS_2 (screen printed)	Origami	30 K	/	/	13.42	/	/	1.59	[26]
BiTe-based TE elements (screen printed)	Origami and Kirigami	90 K	/	/	~0.0625	/	/	0.314	[30]
$Bi_{0.3}Sb_{1.7}Te_3/Bi_2Te_3$ TE blocks	Origami and Kirigami	80 °C	/	/	/	44.7	/	/	[31]
Bi_2Te_3/Sb_2Te_3 (screen printed)	Kirigami	50 °C	/	/	/	in the order of hundreds of pW at 50°C	/	/	[32]
p-Sb_2Te_3 /n-Bi_2Te_3 (screen printed)	Kirigami	60 °C	201	86.451	/	7.64	3.293	/	[33]
$Bi_{0.3}Sb_{1.7}Te_3/Bi_2Te_3$ (TE blocks)	Kirigami	100 °C	8	/	/	~210	/	/	[34]
Bi_2Te_3-Sb_2Te_3 (screen printed)	Origami	75 K	190.7	/	/	~0.08	/	/	[35]
Bi_2Te_3 (screen printed)	Kirigami	55 K	70.5	/	/	~0.596	/	/	[23]
$Bi_2Te_{2.7}Se_{0.3}$/ $Bi_{0.5}Sb_{1.5}Te_3$ (screen printed)	Origami	50 K	229.06 **(plane)** 115.18 **(cylindrical)**	978.91 **(plane)** 492.24 **(cylindrical)**	19.58 **(plane)** 9.84 **(cylindrical)**	11.2 **(plane)** 2.81 **(cylindrical)**	48 **(plane)** 12 **(cylindrical)**	0.96 **(plane)** 0.24 **(cylindrical)**	Our Work
